# Identification of host-microbe interaction factors in the genomes of soft rot-associated pathogens *Dickeya dadantii* 3937 and *Pectobacterium carotovorum* WPP14 with supervised machine learning

**DOI:** 10.1186/1471-2164-15-508

**Published:** 2014-06-21

**Authors:** Bing Ma, Amy O Charkowski, Jeremy D Glasner, Nicole T Perna

**Affiliations:** Genome Center of Wisconsin, University of Wisconsin-Madison, Madison, WI 53706 USA; Department of Plant Pathology, University of Wisconsin-Madison, Madison, WI 53706 USA; Department of Genetics, University of Wisconsin, Madison, WI 53706 USA; Institute for Genome Sciences, University of Maryland School of Medicine, Baltimore, MD 21201 USA

**Keywords:** Pattern recognition, Data mining, Plant pathogen, Genome scale analysis, Enterobacteria

## Abstract

**Background:**

A wealth of genome sequences has provided thousands of genes of unknown function, but identification of functions for the large numbers of hypothetical genes in phytopathogens remains a challenge that impacts all research on plant-microbe interactions. Decades of research on the molecular basis of pathogenesis focused on a limited number of factors associated with long-known host-microbe interaction systems, providing limited direction into this challenge. Computational approaches to identify virulence genes often rely on two strategies: searching for sequence similarity to known host-microbe interaction factors from other organisms, and identifying islands of genes that discriminate between pathogens of one type and closely related non-pathogens or pathogens of a different type. The former is limited to known genes, excluding vast collections of genes of unknown function found in every genome. The latter lacks specificity, since many genes in genomic islands have little to do with host-interaction.

**Result:**

In this study, we developed a supervised machine learning approach that was designed to recognize patterns from large and disparate data types, in order to identify candidate host-microbe interaction factors. The soft rot Enterobacteriaceae strains *Dickeya dadantii* 3937 and *Pectobacterium carotovorum* WPP14 were used for development of this tool, because these pathogens are important on multiple high value crops in agriculture worldwide and more genomic and functional data is available for the Enterobacteriaceae than any other microbial family. Our approach achieved greater than 90% precision and a recall rate over 80% in 10-fold cross validation tests.

**Conclusion:**

Application of the learning scheme to the complete genome of these two organisms generated a list of roughly 200 candidates, many of which were previously not implicated in plant-microbe interaction and many of which are of completely unknown function. These lists provide new targets for experimental validation and further characterization, and our approach presents a promising pattern-learning scheme that can be generalized to create a resource to study host-microbe interactions in other bacterial phytopathogens.

**Electronic supplementary material:**

The online version of this article (doi:10.1186/1471-2164-15-508) contains supplementary material, which is available to authorized users.

## Background

Interactions between plant-associated microbes and their eukaryotic hosts are complex biological processes involving hundreds, if not thousands, of genes from each organism. Understanding the molecular mechanisms of such complex processes at the systems-scale is seriously hampered by the lack of a comprehensive list of gene products that contribute for even a single bacterial or fungal pathogen. Variation in lifestyles and pathogenic potential between organisms makes the challenge all the greater. Genome sequencing has dramatically increased the potential for large-scale screens to identify genes involved with host-microbe interactions. Direct experimental evidence is the obvious gold standard, but not all significant pathogens are experimentally tractable, and selection of experimental conditions and convenient hosts for high-throughput screens can limit discovery. More targeted experiments can be designed to probe function more completely, but these are time consuming and generally limited to a smaller number of candidate genes. Further, it is unclear what experiments to conduct if a candidate gene is of completely unknown function. Importantly, genes of unknown function make up a substantial fraction of each sequenced genome, and it is likely that among these lie some of the greatest potential for discovery of truly novel aspects of host-microbe interaction (as well as many other complex biological processes).

Computational approaches to identify potential host-microbe interaction factors and predict their specific functions can be a valuable way to guide experimentation, and may be the only option for some recalcitrant organisms. Typical bioinformatics strategies include searching for sequence similarity to gene products known to contribute to host-microbe interaction in other organisms, and comparing genomes to identify gene islands that discriminate between pathogens of one type and closely related non-pathogens or pathogens of a different type. Both strategies are useful, but the former is limited to known genes and detectable levels of sequence similarity, and thus excludes the vast collections of genes of unknown function. The latter lacks specificity, since many genes in genomic islands may have little to do with host interactions, and the definition of rules for the distribution across organisms can be arbitrary. There are no simple rules to define the relevant distribution for the set of orthologous genes across genomes, especially when there are a large number of genomes being compared. Further, it is preferable in many situations to factor in other features such as genome context or gene expression data as additional evidence sources to predict whether a gene is associated with host-microbe interaction processes.

More sophisticated computational prediction strategies can introduce a variety of other types of evidence, but integration of diverse data types remains a challenge. Machine learning techniques are ideally suited for pattern recognition tasks to accommodate diverse biological data sources into a single predictive analysis to achieve superior performance over any individual type of data, especially where (1) data sets are large, (2) with heterogeneous sources, and (3) patterns are not easily described by a compact set of rules, all of which are true for the task of genome-scale identification of host-microbe interaction factors. Supervised machine learning schemes have been receiving increasing attention recently as a promising approach to study diverse biomedical problems [1–5], but no previous study focused on host-microbe interaction factors. In this study, we developed a supervised machine learning strategy to identify the gene inventory involved with host-microbe interaction from two soft rot-associated enterobacteria, *Dickeya dadantii* (aka. *Erwinia chrysanthemi*) 3937 [6], and *Pectobacterium carotovorum* (aka. *Erwinia carotovorum*) WPP14 [7]. Our approach allows us to incorporate a wide variety of input data, including homology information, genome context, predicted transcription factor binding sites, and microarray transcript profiles. It has achieved promising results with precision rate over 90% with recall rate over 80%. Further, our study generates an extended list of roughly 200 candidate interaction factors and provides experimentally testable hypotheses to stimulate further research on the molecular mechanisms of soft rot pathogenesis and survival in plant hosts. This study represents a promising application of pattern-recognition methods for identification of factors involved in complex biological processes, which can be generalized to study other plant-associated organisms.

## Methods

### Target genome selection

Soft rot-associated enterobacteria are economically important pathogens that infect a broad range of plant species [8–11]. Soft rot bacterial pathogenesis is characterized by rapid necrosis of parenchymatous tissues, mainly due to the action of secreted enzymes that degrade the middle lamellae and the primary cell wall [12]. Continuing discovery of additional genes involved in survival in a plant host or which contribute directly to pathogenesis [13–19] suggests that even for well-studied organism such as Dd3937, we have not yet achieved a comprehensive list of host-microbe interaction factors or a complete understanding of their precise roles. In this study, we target two soft rot-associated phytopathogens for genome-wide identification of host interaction factors (Table 1). One, *Dickeya dadantii* 3937 (Dd3937) was originally isolated from *Saintpaulia ionantha* [20, 21], and is a long-standing model system for this group of organisms [6]; the other, *Pectobacterium carotovorum carotovorum* WPP14 was isolated from infected potato in Wisconsin [7, 22].

**Table 1:** **Genome-wide target class label assignment to each protein coding gene as a data point for** ***Dickeya didantii*** **3937 and** ***Pectobacterium carotovorum*** **WPP14**

	Total # CDS*	IF**	CF**	Training data set	Testing data set	Pseudogene
**Dd3937**	4520	267	1264	1531	2989	28
**WPP14**	4590	233	1111	1344	3246	174

*we only use protein coding genes and pseudogenes are not included.

**IF stands for host-microbe interaction factor; CF stands for genes involved in core biological processes

Colonization and survival in plants requires numerous factors including proteins involved with iron assimilation, protein secretion, exopolysaccharide synthesis, motility, and stress-resistance [23, 24]. Five Gene Ontology terms were identified that partition the majority of the positive class training set data into distinct aspects of host-microbe interactions (Table 2). We included all data points in most of our analyses, but also conducted analyses on the partitions defined by these GO annotations (Additional file 1a and 1b). This allows us to test whether different subsystems contain distinct patterns that can be recognized by our learning schemes, while avoiding subsystems with too few genes to provide sufficient information to train the learning schemes.

**Table 2:** **Ontology for host-microbe interaction, and category assignment genome-wide for data points in** ***Dickeya dadantii*** **(Dd3937) and** ***Pectobacterium carotovora*** **(WPP14)**

GO term and name	Dd3937	WPP14
GO:0052192 movement in environment of other organism involved in symbiotic interaction;	41	41
GO:0052048 interaction with host via secreted substance involved in symbiotic interaction	54	53
GO:0051816 acquisition of nutrients from other organism during symbiotic interaction	103	81
GO:0044413 avoidance of host defenses	43	34
GO:0043903 regulation of symbiosis, encompassing mutualism through parasitism	13	9
*GO:0044403 symbiosis, encompassing mutualism through parasitism	13	15
Total	267	233

*this term is a parent term for all others listed in this table and is used as a generic catch all for host-microbe interaction factors lacking more specific GO term annotations.

### Assembling training datasets

The data set for each target genome is assembled separately. Genome sequences, predicted proteins and annotations for both genomes were obtained from the ASAP database [25, 26]. Each protein-coding gene in a target genome is considered a data point. The target class label in this specific learning task indicates whether or not a data point has an association with the biological processes involved in host-microbe interaction. A positive class label means the data point is related to host-microbe interaction. A negative class label indicates the data point is not likely to be directly involved in host-microbe interaction, rather it is associated with core biological processes such as transcription and translation or central pathways of metabolism. Positive and negative class labels were assigned by human experts.

For each data point, we assemble a vector of features (or attributes), to characterize it. In our preliminary analyses, we sought to be inclusive in construction of the data matrix. We included 606 attributes for Dd3937 and 598 attributes for WPP14, and these attributes fall roughly into four different categories listed in Table 3. (1) Sequence homology data was obtained from BLASTP searches of the proteins from the target genomes against 239 gamma-proteobacteria from 14 bacterial orders and 58 genomes from other bacterial families outside of gamma-proteobacteria (details in Additional file 2a and 2b). 2) We further summarized sequence homology information by classifying organisms based on phenotypes (e.g.., strict anaerobe), taxonomy (e.g.., the order of *Enterobacteriales*), habitat (e.g., aquatic), and host type (e.g. plant-associated). Based on this information, we calculated a series of attributes summarizing the homology data. For instance, for each gene, we calculate the number of genomes with a homolog, the fraction of genomes with homologs that are plant-associated, the average similarity scores between homologs, the ratio of the similarity score of plant-associated versus animal-associated homologs, the percentage of hits in the order of *Enterobacteriales*, and the percentage in facultative anaerobic organisms, etc. Additional file 2c shows the number of genomes in each category used to generate summary attributes. 3) Information related to function and regulation including transcriptome and proteome profiles was incorporated into the attribute vectors (details in Additional file 3a), including microarray experiments with a *pecS* mutant strain [27], exposure to phenolic acids [28], and growth on potato tuber and stem [29]. For Dd3937, we also integrated the presence of predicted binding sites for 32 transcriptional regulators, including ones related to gene regulation during infection such as PecS [17, 27], KdgR [30], H-NS [31, 32], and CRP [33, 34]. We did not include binding site data for WPP14 because the large number of contigs complicates prediction. 4) Finally, we incorporated over 20 basic gene or protein features (Table 3), such as GC content, amino acid composition and computed structural and physiochemical features of proteins and peptides [35], operon prediction [36], COG functional category [37], and codon adaptation index [38, 39]. Other gene features are derived from more complex analyses, including: (a) the phylogenetic profile method [40], which is based on the theoretical framework that co-occurrence of functionally linked proteins will be preserved by natural selection [41]; (b) Phylogenetic conservation which classifies genes according to distribution at different branching depths based on our phylogenetic framework for enterobacteria [11]; (c) PSORTb v3.0 [42] which predicts localization as cytoplasmic, cytoplasmic membrane, periplasmic, extracellular, or unknown; (d) Protein fingerprint scanning (a similarity search technique able to identify distantly related proteins) against identified fingerprints associated with virulence factors in PRINTS database [43, 44]; and (e) the gene neighbor method which identifies gene physical adjacency on a chromosome [45], based on the theory that neutral evolution tends to shuffle gene orders while functionally associated genes have conserved gene order. We employ both 150 bp and 300 bp as a threshold distance to define gene neighbors using *ad hoc* code.

**Table 3:** **List of all attributes categories used in data set formation in this study, and number of attributes in each categories for all data points in training data set for** ***Dickeya dadantii*** **(Dd3937) and** ***Pectobacterium carotovorum*** **(WPP14)**

Category	Subcategory	Dd3937	WPP14	Reference
**Sequence homology**	***Subtotal***	**297**	**297**	
	Gamma strains	239	239	Additional file 2a
	Non-gamma strains	58	58	Additional file 2b
**Phenotypes of interest**	***Subtotal***	**194**	**194**	
	Taxonomy Statistics	76	76	Additional file 2c, d
	Lifestyle Statistics	118	118	Additional file 2c, d
**Gene characteristics**	***Subtotal***	**23**	**21**	
	GC content	1	1	This study
	subcellular localization	1	1	[42, 46]
	phylogenetic profile	6	6	[40, 41]
	fingerprints scanning	3	3	[43, 44]
	codon adaptation index (CAI)	3	3	[47, 48]
	physical adjacency (gene neighbor)	2	2	[49, 50]
	Operon prediction	1	1	[36, 51]
	phylogenetic conservation	1	1	This study
	COG functional category	1	1	[52]
	Genomic island	4	1	[53, 54]
	computed structural and physicochemical features of proteins and peptides	40	66	[35, 55]
**Functional genomics**	***Subtotal***	**52**	**3**	
	binding site prediction	32	0	Additional file 3b
	Gene expression	14	3	Additional file 3a
	proteomics	6	0	Additional file 3a
	**Total**	**606**	**581**	

### Overview of supervised machine learning procedures

**Figure 1:**
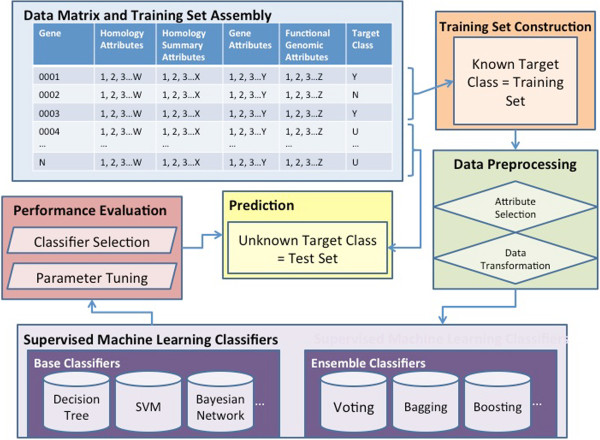
**Flow chart of the procedures in performing supervised machine learning tasks of host-microbe interaction factor prediction.**

The learning procedure is illustrated in Figure 1. (1) First training and testing data sets are assembled by assigning target class labels and forming attribute vectors. (2) Data preprocessing is performed to improve representation and quality, including attribute selection and data transformation, as well as data partitioning according to GO annotations. (3) Both data preprocessing and pattern learning schemes were implemented in Weka package version 3.5.6. [56, 57]. Both base and ensemble classifiers were trained to recognize classification patterns. Seven base classifiers were employed in this study including decision tree [58], support vector machine (SVM) using sequential minimal optimization [59–61], Bayesian probabilistic approaches including Bayesian network [62, 63] and naive bayes [64], instance based learner k nearest neighbor [65], and propositional rule learner using repeated incremental pruning to produce error reduction (RIPPER) [66]. On top of base classifiers, ensemble classifiers, such as bagging and boosting classifiers, combine multiple models by either sub-sampling a given dataset to achieve greater predictive accuracy and reduce overfitting bias [67–70] or combining of probability estimates from different methods [71–73]. Detailed algorithm descriptions and specific settings are described in Additional file 4. (4) Classifier training is followed by classifier performance evaluation, comparison, and selection. Cross-validation is a technique to assess how accurately a predictive model will perform on an independent data set and whether the model recognizes a pattern that is generalized enough to apply to unseen data [74, 75]. (5) Based on performance on the training set, we selected the best classifiers to build models and make predictions for the genes that were not part of the training sets.

### Data preprocessing

Attribute extraction, or data transformation, was used to improve the representation of the data sets. Data transformation techniques create extracted attributes from the original attributes, in order to normalize so different attributes are on the same approximate scale, transform all numeric attributes in the dataset to have zero mean and unit variance [76], perform linear mapping of the data to a lower dimensional space in such a way that the variance of the data is maximized using principal components analysis (PCA) [77], or combine attributes where the aggregate feature is more useful than keeping them separate. Since many attributes used in our analysis are continuous data, we also employed data discretization techniques that convert continuous features to discretized or nominal ones to accommodate both data types in the same analysis [78, 79]. Another important component in data preprocessing is attribution selection, which is removal of uninformative data since excessive dimensionality can reduce the effectiveness of learning tasks. It includes two steps: an initial clean-up step where the attributes of each type (as listed in Table 3) are tested individually in order to remove the ones with insignificant contribution to classification, which is especially useful for the data types with highest dimensionality. The second step is to evaluate the importance of an attribute passed on from the initial step, and to remove the ones with low importance measurement scores. We used random forest attribute importance measures in this step, which are based on the decrease of classifier performance when values of a variable in a bifurcating tree node are permuted randomly [80], implemented in the extended version of weka 3.5.1 [81, 82] (More details in Additional file 4). Furthermore, we performed data decay analysis to define compact attribute sets that maintain informativeness. This involved ranking all attributes based on importance measures from 100 runs using random forest classifiers, gradually decreasing the number of attributes by window size 10 based on their rank, recording the performance of all decayed data sets, and defining the essential set as the point where the overall performance score began to drop.

### Evaluating the performance of different learning schemes

We used 10-fold cross-validation analyses to evaluate the learned classifiers on random subsets of data withheld from the training sets and averaged across multiple replicates. We recorded a variety of performance statistics for each run including accuracy, true positive rate (TPR or recall), and precision for the positive target class. We also used ROC (Receiver Operating Characteristic) curves, PR (Precision-Recall) curves, and the AUC (area under the curve) to evaluate the performance of each constructed classifier. In this particular learning task, we value precision rate as the most important statistic. Precision specifies the proportion of relevant objects being retrieved among all retrieved ones, a factor that is particularly important to define a candidate list with high confidence for downstream experimental validation. On the other hand, recall is the proportion of relevant objects that are retrieved. When a situation does not allow both precision and recall rates to be high at the same time, we give the precision rate precedence over the recall rate. ROC and PR curves are widely regarded as more appropriate than any individual statistic in evaluating classification algorithms [83]. A ROC curve is a graphical technique that plots the correlation of correctly classified data points with falsely classified ones, in order to characterize the tradeoff between true positive and false positive rates. PR curves depict the correlation of how precisely the algorithm identifies the data points in their class with how many “true” data points are retrieved and provide a good complement to ROC curves which can be overly optimistic [84].

## Results and discussion

Many computational methods have been used to identify gene functions involved in host-microbe interaction, and most of them rely primarily on homology-based searches using known interaction determinants as bait to identify new candidate genes. These methods are often successful, but neglect many genes of unknown function and strain/clade-specific genes, which could play an important role in host-microbe interactions and bacterial niche adaptations [85, 86]. Overcoming these limitations with the current methodologies is critical to expanding our understanding of the complex molecular mechanisms underlying host-microbe interactions. The value of machine learning not only lies in deriving knowledge based on pattern recognition, but also providing an automated alternative to having a human expert repeatedly sift through large and complex datasets.

### Some attributes are more useful than others to predict host-microbe interaction

**Figure 2:**
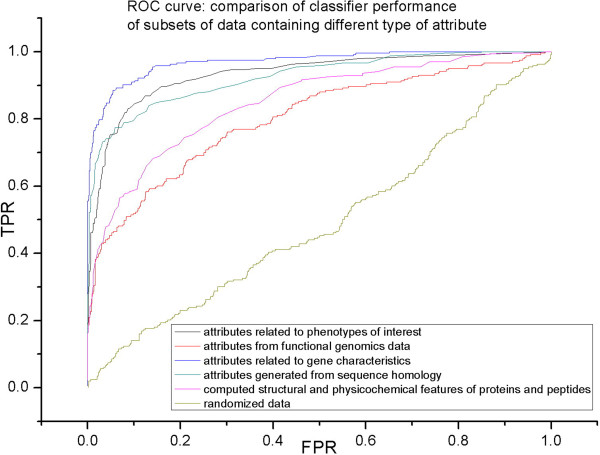
**ROC curve to compare classifier performance of different data sets containing various types of attributes as listed in Table** 3 **.** (TPR: True Positive Rate; FPR: False Positive Rate).

Our results indicate that although all categories outperform randomized data, different major categories of attributes contribute differently to learning scheme performance as shown in the ROC curve for Dd3937 in Figure 2 and Additional file 4. Gene features and summarized homology information were most useful in classifying host-microbe interaction factors, while data related to computed structural or physiochemical characteristics, and gene functionality data, including gene expression, binding site predictions, and proteomics profiles, performed less well. Further analysis of the gene functionality attributes using random forest importance measurement scores indicates that the data corresponding to many of these attributes are relatively noisy and do not correlate well with the target class, though a subset, such as KdgR binding site predictions, do correlate well. Some of our attributes are themselves the results of other pattern recognition methods. For example, phylogenetic profiles, one of the most useful attributes, are based on an unsupervised learning approach, where no prior information is given to the learner regarding the output or class label. Our analysis is a good example of how supervised and unsupervised learning algorithms can be combined to make better inference.

We conducted data decay analysis to obtain additional insight into the most informative attributes. The size of the final compact attribute sets is 45 and 31 for Dd3937 and WPP14, respectively, as shown in Additional file 5b. The majorities of attributes in the compact sets are summaries of homology data according to phenotypes or computed gene features, and many of the retained attributes are shared between both strains despite the independent machine learning analyses. The common list includes five gene feature attributes including phylogenetic profile, gene cluster from operon prediction, gene neighbor, cellular localization, and amino acid composition. The most informative homology attributes include percentage, average value, or sum value of a given gene having homologous hits with organisms having different pathogenicity and habitat phenotypes. In addition, the homology data summarized by phenotypes related to growth condition and taxonomic groups is also informative including having homologs in anaerobic organisms, facultative anaerobes and their ratio, and having homologs in other gamma-proteobacteria, and enterobacteria, all of which appear in the selected attribute list for both strains.

Overall, these results suggest that attributes which are relatively simple to assemble from standard BLASTP searches, coupled with a handful of additional easily computed features are sufficient to achieve good performance in this machine learning task. This is particularly encouraging for development of a generalized approach for future applications to predict host-interaction factors across a broad range of bacterial phytopathogens.

### Preprocessing and partitioning can improve performance

**Figure 3:**
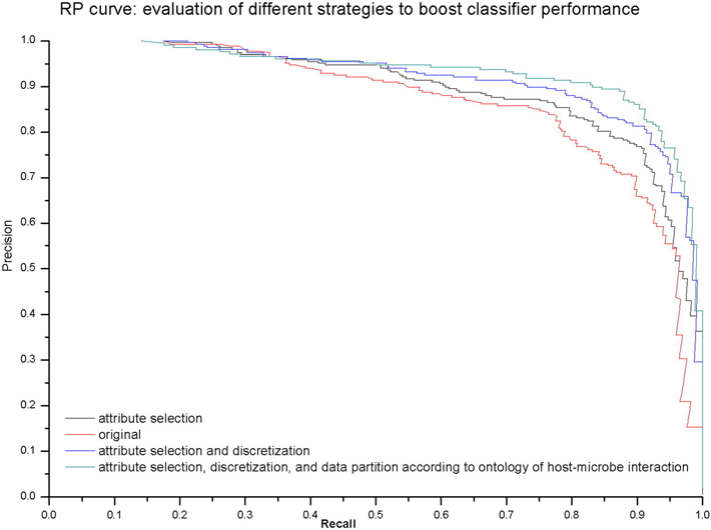
**PR (Precision-Recall) curve to evaluate strategies for boosting classifier performance.**

The PR curve shown in Figure 3 illustrates the improvement in performance that we achieved through attribute selection, data discretization, and data partitioning according to GO terms. 1) Attribute selection generates more cost-effective learning schemes by reducing data set dimensionality by removing uninformative attributes, in order to improve the overall performance of the learning schemes [87, 88]. After benchmarking different attribute selection techniques such as filter (e.g., subset attribute selection [89]) and wrapper methods (e.g., Naive Bayes with forward selection algorithm) as well as attribute ranking (e.g., SVM Attribute evaluator [90] and information gain), we chose random forest importance measures in this study because it is robust to noise, relatively computationally efficient, and is suitable for data sets with high dimensionality hence reducing the risk of overfitting [81]. After feature selection, our data sets contain 105 and 122 attributes, which are 17.3% and 21% of the original data size of Dd3937 and WPP14, respectively. 2) By comparing different data transformation techniques (Additional file 6a), supervised data discretization was shown to be substantially better for improving classifier performance than other methods. Supervised discretization techniques are suitable for high dimensional data as they significantly reduce the number of possible values of continuous features, and also discretize an attribute according to its class label [91, 92]. 3) We also saw an improvement when we coupled the preprocessing with partitioning the learning task into several separate tasks based on assigning genes in the training set according to GO terms. This result suggests that some subsystems, such as localization in host and secretion of host interaction proteins, are substantially more informative and suitable for our learning task (Additional file 6b). Other subsystems, such interaction with host defense systems and transcriptional regulation of host interaction genes, performed less convincingly, possibly because these subsystems are involved in host-microbe interaction but also include other genes not implicated in this biological process. For example, the global DNA-binding regulator *hns* gene also modulates flagella genes and lipopolysaccharide production that are important for initial bacterial attachment to host cell surfaces [93, 94]. These data points were removed from subsequent analysis. Our result suggests that our learning schemes hold predictive power for the subsystems involved with complex biological processes during host-microbe interaction, but do not accurately distinguish the patterns for some subsystems that are closely intertwined with other cellular processes.

### The performance of machine learning schemes is statistically encouraging

In this study, we employed several strategies to mitigate the potential overfitting issues that are important for effective supervised machine learning tasks. Simply put, overfitting occurs when the predictive model learns a pattern that is overly specific to the training data but not generalized enough to perform equally well on unseen data [95]. We strived to maximize inclusion of relevant attributes to mitigate the problem of overfitting to increase model replicability [96], while excluding unimportant attributes that may be detrimental to pattern recognition schemes performance. Additionally, we hold out pristine examples for testing, integrated result over multiple classifiers retaining only predictions that show a high degree of consensus, chose classifier parameters based on the cross-validation tests, and used a simpler predictor where possible, to address the overfitting issue.

**Figure 4:**
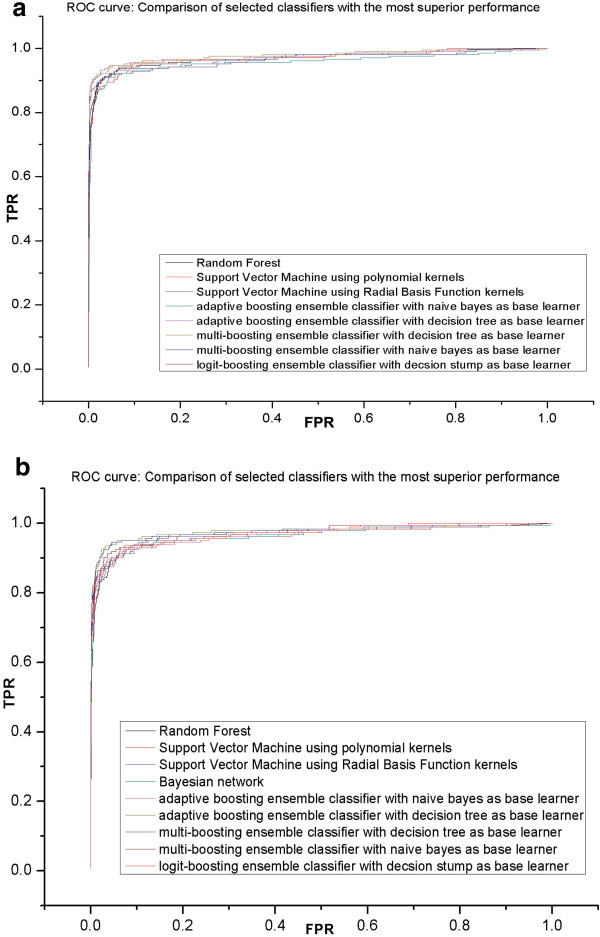
**Comparison of the selected learning schemes. (a)** ROC curve for *Dickeya dadantii* 3937, **(b)** ROC curve for *Pectobacterium carotovorum* WPP14. (TPR: True Positive Rate; FPR: False Positive Rate).

Overall the results of using supervised machine learning schemes on host-microbe interaction factor prediction are statistically encouraging, achieving over 84% precision rate and 75% recall rate from 10-fold cross validation evaluation. We used a nested 10-fold cross-validation that includes an “outer” 10-round cross-validation, which averages data variability from 10 different data partitions. Each data partition sets aside 10% of the data set (outer test set) to measure the performance of the predictive model generated from the other 90% of the data (outer training set). Each outer training set is used to choose the value of tuning parameters for this model in order to achieve optimal performance. The parameter-tuning step is especially important for SVM and K-nearest neighbor learning schemes which are particularly sensitive to parameter settings (Stone 1977). Performance statistics for different classifiers are listed in Table 4, excluding classifiers with precision rates < 80%. ROC curves of selected classifiers for WPP14 are shown in Figure 4.

**Table 4:** **Statistics for positive class object prediction and parameters used in selected learning schemes for both** ***Dickeya dadantii*** **3937 and** ***Pectobacterium carotovorum*** **WPP14**

Classifiers	Precision	TPR/recall/sensitivity	specificity/TNR	accuracy	F-measure	AUC
**Dd3937**						
Random Forest	0.93	0.81	0.98	0.94	0.87	0.97
Bayesian Network	0.91	0.85	0.97	0.94	0.88	0.97
SMO using RBF kernels	0.93	0.85	0.98	0.95	0.89	0.92
SMO using polynormial kernels	0.91	0.87	0.97	0.95	0.95	0.89
Adaptive Boosting (Naïve Bayes)*	0.84	0.89	0.95	0.93	0.87	0.96
Adaptive Boosting (Decision Tree)*	0.96	0.91	0.99	0.97	0.93	0.98
Adaptive Boosting (IBK)*	0.96	0.84	0.99	0.95	0.90	0.99
Adaptive Boosting (Decision Stump)*	0.92	0.87	0.98	0.95	0.89	0.97
Multi-Boosting (Decision Tree)*	0.97	0.91	0.99	0.97	0.94	0.98
Multi-Boosting (IBK)*	0.91	0.77	0.98	0.93	0.84	0.93
Multi-Boosting (Naïve Bayes)*	0.90	0.91	0.97	0.95	0.91	0.96
Logit-Boosting (Decision Stump)*	0.91	0.90	0.97	0.96	0.91	0.98
**WPP14**						
Random Forest	0.89	0.81	0.97	0.93	0.85	0.97
Bayesian Network	0.90	0.83	0.97	0.94	0.87	0.97
SMO using RBF kernels	0.94	0.84	0.98	0.95	0.89	0.91
SMO using polynormial kernels	0.93	0.86	0.98	0.95	0.95	0.89
Adaptive Boosting (Naïve Bayes)*	0.89	0.89	0.97	0.95	0.89	0.96
Adaptive Boosting (Decision Tree)*	0.95	0.86	0.99	0.96	0.90	0.98
Adaptive Boosting (IBK)*	0.87	0.83	0.96	0.93	0.85	0.92
Logit-Boosting (Decision Stump)*	0.90	0.85	0.97	0.94	0.88	0.97
Multi-Boosting (Decision Tree)*	0.94	0.86	0.98	0.96	0.90	0.98
Multi-Boosting (Decision Stump)*	0.91	0.75	0.98	0.93	0.82	0.97
Multi-Boosting (Naïve Bayes)*	0.90	0.89	0.97	0.95	0.89	0.96
Logit-Boosting (Decision Stump)*	0.90	0.87	0.97	0.95	0.89	0.97

*: denote ensemble classifiers, with base learner being shown within parenthesis.

*Abbr:* SMO: Support Vector Machine using Sequential Minimal Optimization; IBK: instance based learner with K-nearest neighbor classifier; RBF: Radial Basis Function kernels.

The comparison of base classifier performances indicates SVM and random forest outperforms other base classifiers (data not shown), and ensemble classifiers generally perform better than base classifiers, especially the boosting algorithms using decision trees as the base learner. The ensemble classifiers integrate results over multiple classifiers in order to average out the “classifier effect”. For example, some classifiers such as Naïve Bayes can be overly optimistic with a lower precision rate [97], and adaptive boosting ensemble classifiers with Naïve Bayes as the base learner can optimize precision and total accuracy rate through incrementally iterative learning processes [98]. The performance curves of selected classifiers are shown in Figure 4a and 4b for Dd3937 and WPP14 respectively. The best performing classifier for Dd3937 is the adaptive boosting ensemble classifier [70] with decision trees as the base learner, which achieved a precision rate above 97% with over 87% recall rate. The best performing classifier for WPP14 is the multi-boosting ensemble classifier [69] with decision trees as the base learner, which reached a precision above 94% with over 82% recall rate. Using the constructed predictive models from selected classifiers, we are able to make predictions for data points with previously unknown relation to host-microbe interactions.

### A significantly extended list of host-microbe interaction factors is revealed

Application of different learned classifiers to the target genomes as a whole allows us to generate a conservative set of predictions for downstream experimentation. We pay the most attention to precision to ensure the retrieved data points are most relevant to host-microbe interaction to facilitate subsequent experimental validation. In order to call a gene a “predicted host-interaction factor”, we required strict consensus across the different classifiers with an average precision score in excess of thresholds defined by the ROC curves (92% and 89% for Dd3937 and WPP14, respectively). The selected classifiers generally agree with each other, and about two thirds of all unknown genes are unanimously predicted by all classifiers to be either host-microbe interaction factors or genes involved in core biological processes. Using these criteria, a total of 1726 genes (57.7% of Dd3937 genes) in Dd3937 and 2180 genes (67.2% of WPP14 genes) in WPP14 are predicted not to involved in host-microbe interactions. There are 211 genes (7.1% of Dd3937 genes) in Dd3937 and 216 genes (6.7% of WPP14 genes) in WPP14 classified as putative interaction factors. The remaining 1052 genes (35.1% of Dd3937 genes) and 850 genes (26.2% of WPP14 genes) are left as unclassified. The top 50 predicted host-microbe interaction factors for *Dickeya dadantii* 3937 and *Pectobacterium carotovorum* WPP14 are listed in Tables 5, 6 and 7, and the entire list of predicted host-microbe interaction factors for both strains are in Additional file 7a and 7b. These lists partially overlap, with 56 orthologs identified as interaction factors in both organisms. Given the phylogenetic relationship between these two phytopathogens and the similarity of their pathogenic phenotypes, we did expect this result; however, the learning tasks were executed independently and agreement across organisms was not a given.

**Table 5:** **Top 50 predicted host-microbe interaction factors from** ***Dickeya dadantii*** **3937**

FeatureID	Prob	Name	Annotation
ABF-0018715	0.922	virB8	Inner membrane protein forms channel for type IV secretion of T-DNA complex (VirB8)
ABF-0020188	0.922		Predicted cell-wall-anchored protein SasA (LPXTG motif) this is up-regulated by hrpY; we have a mutation in this gene.
ABF-0019950	0.922		Putative multicopper oxidase
ABF-0019360	0.922		hypothetical protein
ABF-0019151	0.922		chrysobactin synthetase cbsF
ABF-0019124	0.922		Biopolymer transport protein ExbD/TolR
ABF-0019122	0.922		MotA/TolQ/ExbB proton channel family protein
ABF-0019117	0.922	sftP	TonB-dependent receptor
ABF-0019116	0.922		hypothetical protein
ABF-0018783	0.922		putative transmembrane protein
ABF-0018775	0.922		Holin
ABF-0018724	0.922		putative ATP/GTP-binding protein remnant
ABF-0018722	0.922	virB2	Major pilus subunit of type IV secretion complex (VirB2)
ABF-0047137	0.922		hypothetical protein
ABF-0018717	0.922	virB6	Integral inner membrane protein of type IV secretion complex (VirB6)
ABF-0018716	0.922	virB7	TriF protein
ABF-0018713	0.922	virB10	Inner membrane protein forms channel for type IV secretion of T-DNA complex (VirB10)
ABF-0018712	0.922	virB11	ATPase provides energy for both assembly of type IV secretion complex and secretion of T-DNA complex (VirB11)
ABF-0018601	0.922		hypothetical protein
ABF-0018207	0.922		hypothetical protein
ABF-0018199	0.922	ganC	putative truncated PTS system EIIBC component
ABF-0017777	0.922	hecA2	Putative member of ShlA/HecA/FhaA exoprotein family
ABF-0015606	0.922		ABC transporter permease protein
ABF-0015604	0.922		Amino acid ABC transporter, periplasmic amino acid-binding protein
ABF-0015543	0.922		hypothetical protein 15544 is up-regulated by hrpY. Is 15543 in the same operon? We have a mutation in 15544
ABF-0015387	0.922	nipE	necrosis-inducing protein
ABF-0014838	0.922		putative exported protein
ABF-0014623	0.922		Type IV pilus biogenesis protein PilN
ABF-0018720	0.922	virB4	ATPase provides energy for both assembly of type IV secretion complex and secretion of T-DNA complex (VirB4)
ABF-0018714	0.922	virB9	VirB9
ABF-0047204	0.922		hypothetical protein
ABF-0015913	0.921	ppdA	Prepilin peptidase dependent protein A
ABF-0017252	0.921		Conjugative transfer protein TrbG
ABF-0018195	0.921	ganG	galactan ABC transport system, permease component
ABF-0016407	0.921		hypothetical protein
ABF-0018205	0.921		Pirin
ABF-0019418	0.921		Cellulose 1, 4-beta-cellobiosidase precursor
ABF-0019468	0.921		hypothetical protein
ABF-0019566	0.921		hypothetical protein
ABF-0016680	0.921		Iron utilization protein
ABF-0020727	0.921	sttG	General secretion pathway protein G
ABF-0019115	0.921		hypothetical protein
ABF-0015381	0.921	avrM	Avirulence protein
ABF-0018723	0.921	virB1	VirB1
ABF-0015598	0.921		hypothetical protein
ABF-0015609	0.921		Branched-chain amino acid aminotransferase
ABF-0018193	0.921	ganF	galactan ABC transport system, permease component
ABF-0017097	0.921		Methyl-accepting chemotaxis protein
ABF-0020433	0.921		hypothetical protein
ABF-0019153	0.921	cbsH	chrysobactin oligopeptidase CbsH

**Table 6:** **Top 50 predicted host-microbe interaction factors from** ***Pectobacterium carotovorum*** **WPP14**

ID	Prob	Name	Product
ADT-0001591	0.912		hypothetical protein
ADT-0003750	0.912		putative exported protein
ADT-0000805	0.912	dltB	peptidoglycan biosynthesis protein
ADT-0003928	0.911		pectate lyase
ADT-0003247	0.911		methyl-accepting chemotaxis protein
ADT-0000806	0.911	dltD	poly(glycerophosphate chain) D-alanine transfer protein
ADT-0003745	0.911		ABC transporter ATP binding protein
ADT-0002063	0.911		hypothetical protein
ADT-0000400	0.911	hasE	HlyD family secretion protein
ADT-0003089	0.911		N-terminal fragment of a diguanylate cyclase (pseudogene)
ADT-0003418	0.910		methyl-accepting chemotaxis protein
ADT-0000941	0.910		methyl-accepting chemotaxis protein
ADT-0006368	0.910		hypothetical protein
ADT-0005582	0.910		hypothetical protein
ADT-0000983	0.910		methyl-accepting chemotaxis protein
ADT-0001252	0.910		ABC transporter permease protein
ADT-0003245	0.910		methyl-accepting chemotaxis protein
ADT-0000027	0.910		methyl-accepting chemotaxis protein
ADT-0003542	0.909		putative type IV pilus protein
ADT-0001195	0.909		LysR-family transcriptional regulator
ADT-0004315	0.909	astB	sulfate ester ABC transporter permease protein
ADT-0003152	0.909		methyl-accepting chemotaxis protein
ADT-0002357	0.908		methyl-accepting chemotaxis protein
ADT-0000543	0.908		ABC transporter, substrate binding protein
ADT-0001392	0.908		putative exported protein
ADT-0002087	0.908		putative signaling protein
ADT-0001868	0.908		LysR-family transcriptional regulator
ADT-0000803	0.908		acyl carrier protein
ADT-0000571	0.908		putative cellulase
ADT-0000535	0.908		putative lipoprotein
ADT-0001404	0.907		hypothetical protein
ADT-0004320	0.907	sftP	TonB-dependent receptor
ADT-0001744	0.907		putative exported protein
ADT-0003391	0.907		putative membrane protein
ADT-0003535	0.907		hypothetical protein
ADT-0003563	0.907		LysR-family transcriptional regulator
ADT-0001980	0.907		hypothetical protein
ADT-0000804	0.907	dltA	putative D-alanine--poly(phosphoribitol) ligase subunit 1
ADT-0001616	0.906		putative transport system membrane protein
ADT-0001394	0.906		hypothetical protein
ADT-0001320	0.906		methyl-accepting chemotaxis protein
ADT-0001567	0.906		putative exported protein
ADT-0005614	0.906		hypothetical protein
ADT-0001436	0.906		putative component of polysulfide reductase
ADT-0004253	0.906	occQ	octopine transport system permease protein
ADT-0001493	0.905		hypothetical protein
ADT-0001492	0.905		putative lipoprotein
ADT-0002704	0.905		putative lipoprotein
ADT-0002584	0.905		ABC transporter, membrane spanning protein

**Table 7:** **List of 56 genes predicted host-microbe interaction factors in both** ***Dickeya dadantii*** **3937 and** ***Pectobacterium carotovorum*** **WPP14**

Dd3937			WPP14		
FeatureID	Name	Product	FeatureID	Name	Product
ABF-0019117	sftP	TonB-dependent receptor	ADT-0004320	sftP	TonB-dependent receptor
ABF-0019116		hypothetical protein	ADT-0004318		unknown
ABF-0018207		hypothetical protein	ADT-0001980		hypothetical protein
ABF-0015604		Amino acid ABC transporter	ADT-0000748		putative extracellular solute-binding protein
ABF-0015387	nipE	necrosis-inducing protein	ADT-0000781		putative exported protein
ABF-0014838		putative exported protein	ADT-0002655		putative exported protein
ABF-0019124		Biopolymer transport protein ExbD/TolR	ADT-0002263		putative biopolymer transport protein
ABF-0019115		hypothetical protein	ADT-0002265		hypothetical protein
ABF-0017097		Methyl-accepting chemotaxis protein	ADT-0003418		methyl-accepting chemotaxis protein
ABF-0019566		hypothetical protein	ADT-0001832		putative exported protein
ABF-0016407		hypothetical protein	ADT-0001404		hypothetical protein
ABF-0015906		6-phosphogluconolactonase	ADT-0003106		putative exported protein
ABF-0019118	atsR	Alkanesulfonates-binding protein	ADT-0001174	atsR	putative sulfate ester binding protein
ABF-0019125	astB	Alkanesulfonates transport system permease protein	ADT-0004315	astB	sulfate ester ABC transporter permease protein
ABF-0017125	inh	Alkaline proteinase inhibitor precursor	ADT-0001911	inh	protease inhibitor
ABF-0019002		hypothetical protein	ADT-0001744		putative exported protein
ABF-0019205		ABC transporter	ADT-0002584		ABC transporter
ABF-0014642		hypothetical protein	ADT-0000571		putative cellulase
ABF-0019092		Transcriptional activator protein lysR	ADT-0001195		LysR-family transcriptional regulator
ABF-0016585		Methyl-accepting chemotaxis protein	ADT-0001320		methyl-accepting chemotaxis protein
ABF-0019383		D-alanyl transfer protein DltB	ADT-0000805	dltB	peptidoglycan biosynthesis protein
ABF-0019855		Methyl-accepting chemotaxis protein II (aspartate chemoreceptor protein)	ADT-0001887		putative methyl-accepting chemotaxis protein
ABF-0015168	chmX	Methyl-accepting chemotaxis protein III (ribose and galactose chemoreceptor protein)	ADT-0003152		methyl-accepting chemotaxis protein
ABF-0018737		DNA-binding protein	ADT-0003335		putative regulatory protein
ABF-0019933		hypothetical protein	ADT-0003354		hypothetical protein
ABF-0014645		Paraquat-inducible protein A	ADT-0002701		putative membrane protein
ABF-0017674		Methyl-accepting chemotaxis protein	ADT-0003245		methyl-accepting chemotaxis protein
ABF-0020681		hypothetical protein	ADT-0002418		RES domain-containing protein
ABF-0015907		TonB-dependent hemin	ADT-0002398		TonB-dependent hemin
ABF-0018934		4-aminobutyrate aminotransferase	ADT-0002845		putative class-III aminotransferase
ABF-0014824		Methyl-accepting chemotaxis protein II (aspartate chemoreceptor protein)	ADT-0002104		methyl-accepting chemotaxis protein
ABF-0018178		Iron(III) dicitrate-binding protein	ADT-0002009		putative periplasmic substrate-binding transport protein
ABF-0019391		Pectate lyase	ADT-0003928		pectate lyase
ABF-0015887		hypothetical protein	ADT-0002063		hypothetical protein
ABF-0016115		Methyl-accepting chemotaxis protein	ADT-0000027		methyl-accepting chemotaxis protein
ABF-0019101	atsB	Alkanesulfonates transport system permease protein	ADT-0003749	atsB	putative sulfate ester transporter
ABF-0019214		Glucosamine kinase GpsK	ADT-0003604		hypothetical protein
ABF-0016752		Ferric siderophore transport system	ADT-0003559		TonB-like protein
ABF-0016218		Fosmidomycin resistance protein	ADT-0001196		MFS efflux transporter
ABF-0046571		Putative DNA-binding transcriptional regulatory family of the TetR family	ADT-0003719		TetR-family transcriptional regulator
ABF-0014644		Probable lipoprotein	ADT-0000406		putative lipoprotein
ABF-0015918	ppdC	Putative prepilin peptidase dependent protein	ADT-0002557	ppdC	putative prepilin peptidase dependent protein c precursor
ABF-0018572		ABC transporter	ADT-0001164		putative iron (III) ABC transporter
ABF-0017527		Lysophospholipase	ADT-0001494		putative lipoprotein
ABF-0047106		putative lipoprotein	ADT-0002704		putative lipoprotein
ABF-0016810		Drug resistance transporter	ADT-0001435		putative membrane protein
ABF-0019088		Dihydrodipicolinate synthase	ADT-0002292		putative dihydrodipicolinate synthetase
ABF-0014868		Ferrichrome-iron receptor	ADT-0004187		TonB dependent receptor
ABF-0017095		hypothetical protein	ADT-0000555		putative exported protein
ABF-0018540		Oxidoreductase	ADT-0000962		probable short-chain dehydrogenase
ABF-0014948		hypothetical protein	ADT-0002252		putative exported protein
ABF-0020431		Methyl-accepting chemotaxis protein I (serine chemoreceptor protein)	ADT-0000661		methyl-accepting chemotaxis protein
ABF-0019851		Methyl-accepting chemotaxis protein III (ribose and galactose chemoreceptor protein)	ADT-0001602		methyl-accepting chemotaxis protein
ABF-0020368		hypothetical protein	ADT-0002020		putative exported protein
ABF-0016058		Poly(glycerophosphate chain) D-alanine transfer protein DltD	ADT-0000806	dltD	poly(glycerophosphate chain) D-alanine transfer protein
ABF-0019212		N-Acetyl-D-glucosamine ABC transport system	ADT-0002138		extracellular solute-binding protein

One striking observation is the large number of genes of unknown function from the predicted list of host-microbe interaction factors. Among all predicted interaction factors, over 30% of them currently have no or very little annotated information, and many of them are ORFans [99–102] without any homolog to 297 bacterial genomes inspected. Among the 56 genes found in interaction factor lists for both strains, roughly one third have no clear functional assignment. 13 hypothetical proteins in both strain lists are “unknown unknowns”, a term used to indicate there is no information at all available for that gene [103]. The other 9 of them are so-called “known unknown” proteins, meaning they only have information in general biological terms, such as putative exported protein, putative transmembrane protein, and probable lipoprotein. This result suggests a substantial portion of the genome cannot be screened using conventional similarity-based searches, and our more sophisticated pattern recognition approach was able to identify candidate interaction factors that would be missed using homology-based methods.

The remaining two-thirds of predicted interaction factors are annotated with various (at least partially) informative functions. The lists include genes with previously characterized roles in host-microbe interaction in these or very closely related organisms that were overlooked by the human experts who assembled the training set. For example, Dd3937 secretes plant cell wall degrading enzymes through a type II secretion system for plant host cell wall degradation in turn using the released nutrients as carbon sources for growth [104], and a group of genes related to this process are predicted with high confidence including predicted proteins previously reported to play an accessory role in utilization of galactose, a major component of pectin, in Dd3937 [105]. A knockout mutant of a necrosis-inducing protein included in the prediction list has been experimentally shown to have reduced virulence in a *Pectobacterium* strain [106]. Further, our lists also include genes with homologs implicated in host-microbe interaction in more distantly related organisms. There are 9 genes that were shown with direct or indirect evidence to be involved with metal homeostasis in different bacteria, including *exbB*, *exbD*, and *tonB* genes which are essential for ferric iron uptake in *Escherichia coli* [107], *Xanthomonas campestris* [108], *Pseudomonas putida* [109], and *Photorhabdus temperate* [110], as well as ferric siderophore transporter and ferrichrome-iron receptor genes, and a cytochrome b gene (*cybC*) that is positively regulated by Fur and others that encode iron-dependent proteins in *Salmonella enterica* [111]. The predicted lists also include orthologs of the *dltB* gene implicated in cell surface adhesion in *Staphylococcus aureus* [112], the *srfA* gene that encodes secreted effect or protein in *Pantoea ananatis* [113], a LysR-family regulator associated with quorum sensing in *Pseudomonas aeruginosa* [114], the cell-wall-anchored protein SasA suggested to play a role in adhesion to host in *Staphylococcus aureus* [115], and the *ppdC* gene involved in extracellular secretion machinery in *Pseudomonas aeruginosa* [116]. Additionally, we also observed many predicted interaction factors that are physically clustered together on the chromosome. For instance, our prediction list includes an 11-gene cluster for a general secretion system, and a 12-gene cluster that may be associated with type IV secretion complex formation. This result agrees with previous studies that many virulence properties of microbes are a collaborative effort of multiple genes and their physical clustering (and/or co-expression as operons) is under functional and evolutionary constraints [117, 118].

Interestingly, our predicted host-microbe interaction factor lists include at least 17 chemotaxis or motility associated proteins for each organism, including putative methyl-accepting chemotaxis receptors and one type IV pilus biogenesis protein involved in bacterial motility and adhesion to a solid surface [119]. Previous studies have indicated the chemotactic responses with specific cellular localization are critical for biofilm formation and interaction with hosts in a variety of pathogenic bacteria [120–124]. The hypergeometric distribution was used to assess the statistical significance of enrichment of a given functional group in the target list relative to the genome as a whole [125, 126]. Interpro family annotations were uniformly assigned across both genomes and we conducted enrichment tests based on assignment to the Interpro chemotaxis family. The highly significant p-values for both Dd3937 (p = 3.42e-11) and WPP14 (p = 3.36e-12) strongly suggest methyl-accepting chemotaxis genes are highly enriched among the predicted host-microbe interaction factors.

Our learning strategy was explicitly designed to separate genes likely to be involved in host-microbe interaction from genes involved with core biological processes. The evidence above strongly suggests that the method is effective at recognizing host-microbe interaction factors, but it is important to keep in mind that it does not directly address the possibility that some genes associated with core biological processes may also contribute to interaction with hosts. Direct experimental testing of a relatively large number of genes from both the positive and negative classes is underway and will illuminate the power of this machine learning approach to guide discovery.

## Conclusion

Although bacterial pathogen genome sequencing has become routine, the large number of unknown genes has been, and still is, a major obstacle to understanding the mechanisms of infection and adaptive evolution of microbial pathogens overall. We successfully employed supervised machine learning to identify candidate host interaction factors and we are able to predict host-microbe interaction factors from among genes of entirely unknown function, for two important agricultural pathogens *Dickeya dadantii* Dd3937 and *Pectobacterium carotovorum* WPP14, achieving promising results with a precision rate over 90% with a recall rate over 80%. The predictions made in this study include many genes that have not previously been linked to host microbe interaction, a result not achievable with homology-based search strategies, providing an expanded list of appealing targets for further experimental validation. Our results indicate the learning schemes used in this study can recognize the complex patterns of host-microbe interaction factors and yield biologically meaningful results. Because of the powerful and intelligent models supervised machine learning schemes are capable of constructing, their future application to studying additional complex biological processes is likely to be a productive research approach.

## Availability of supporting data

The data sets supporting the results of this article are available in the LabArchives repository, [https://mynotebook.labarchives.com/share/plantpath/MjAuOHwyNTc2OC8xNi9UcmVlTm9kZS8yNjQ4MTE0NTE0fDUyLjg=].

## Electronic supplementary material

Additional file 1: **List of individual protein-coding genes from Dd3937 (a) and WPP14 (b) assigned host-microbe interaction GO annotations corresponding to Table** 2 **.** (ZIP 33 KB)

Additional file 2: **(a) Strain information of all gamma-proteobacteria with complete or nearly complete genomes used in this study and their associations with various phenotypes of interest (as listed in Additional file 2: Table S1).** (b) List of all non gamma-proteobacteria with complete or nearly complete genomes used in this study. (c) List of attribute categories used to generate summary taxonomic and phenotypic attributes based on sequence homology, the count of genomes in each category used as BLASTP search databases, and the number of proteins in *Dickeya dadantii* (Dd3937) and *Pectobacterium carotovorum* (WPP14) with hits to target organisms in those categories. (d) List of attributes used in the category of phenotypes of interest, including taxonomic groups and lifestyle statistics. (ZIP 223 KB)

Additional file 3: **Functional genomics data sets used in this study.** (a) Data sets generated from microarray or proteomics studies. (b) Binding sites prediction of 32 putative transcriptional factors that may be related with host-microbe interactions. (ZIP 34 KB)

Additional file 4: **Description of selected supervised machine learning schemes.** (DOCX 150 KB)

Additional file 5: **(a) List of selected attributes with importance measurement score and subsets of attributes defined to be used in data decay analysis for** ***Dickeya dadantii*** **3937 and** ***Pectobacterium carotovorum*** **WPP14, (b) probability score plot used to define a compact set of attributes for both strains.** (ZIP 1 MB)

Additional file 6: **(a) RP curve to compare strategies of data transformation for boosting classifier performance.** (b) PR curve to compare classifier performance using five data sets partitioned according to GO terms for different aspects of host-microbe interaction (refer to Table 2). (ZIP 223 KB)

Additional file 7: **The list of predicted host-microbe interaction factors.** (a) List of 211 genes predicted to be host-microbe interaction factors for *Dickeya dadantii* 3937, (b) list of 216 genes predicted to be host-microbe interaction factors for *Pectobacterium carotovorum* WPP14. (c) list of top 300 genes that could not be confidently classified as host-microbe interaction factors for both *Dickeya dadantii* 3937 and *Pectobacterium carotovorum* WPP14. (ZIP 17 KB)

## Abbreviations

RF: Random forest
SMO: Support Vector Machine using Sequential Minimal Optimization
poly: SMO using polynomial kernels
rbf: SMO using Radial Basis Function (RBF) kernels
ab: Adaptive boosting
j48: C4.5 algorithm in decision tree
mb: Multi-boosting
nb: Naive bayes
lb: Logitboost
ds: Decision stump
BN: Bayesian network.
