# Supplementary material for: Identification of host-microbe interaction factors in the genomes of soft rot-associated pathogens Dickeya dadantii 3937 and Pectobacterium carotovorum WPP14 with supervised machine learning
Source: BMC Genomics. 2014 Jun 21;15:508. doi: 10.1186/1471-2164-15-508 (PMC4079955; doi:10.1186/1471-2164-15-508)
Supplement: Supplementary file 4 — Additional file 4: Description of selected supervised machine learning schemes. (DOCX 150 KB) [file 12864_2013_6186_MOESM4_ESM.docx]

**Description of selected supervised machine learning schemes**

As with different strategies used in concept learning ([Bruner, Goodnow, and Austin 1967](#_ENREF_4)), various supervised machine learning schemes differ in how they present the concept to be learned, which is the host-microbe interaction category used to classify genes. In the following we briefly describe a few common learning schemes we included in this study.

K-nearest neighbor:

The K nearest neighbor method classifies a data point based on a majority vote of its nearest neighbors defined by distance (e.g., Euclidian distance) between data points. The predicted class label is the most common label among the data point’s k nearest neighbors. The advantage of using this method is that its result is strongly consistent and easy to interpret, but it is very sensitive to the parameter k (number of neighbors) that needs to be carefully tuned. Data dimensionality can have a considerable impact on its model construction using this method.

Decision tree:

The decision tree algorithm constructs a bifurcating tree-like model, based on which to assign data points to a target class. The tree model is constructed by recursively adding internal tree nodes by selecting the attribute that can split data points into a “purest” subset with the same target class label, which is calculated based on information gain or entropy. An advantage of the decision tree method is that the tree model that is generated can be explicitly explained and visualized. However, the decision tree needs to be carefully “pruned” to prevent it from over-fitting on training data, and because the method depends on a greedy algorithm it may become stuck in a local optimum.

Bayesian classifiers:

Naive bayes methods assume the attributes are conditionally independent, which is clearly not true for some of the attributes under consideration here, and in many other machine learning tasks. Yet, in practice these methods perform reasonably well and very efficiently, though they are also subject to the issue of data dimensionality. Bayesian network approaches theoretically overcome the shortcomings of naive bayes by using a directed acyclic graph (DAG) to represent a conditional probability distribution that describes relationships between features and class of interest. The structure of a Bayesian network is learned by searching through the space of possible networks, and our preliminary results suggested TAN (tree augmented naive bayes) ([Friedman, Geiger, and Goldszmidt 1997](#_ENREF_5)) outperforms other searching algorithms, which included climbing, tabu search that performs hill climbing until it hits a local optimum and then it steps to the least worst candidate in the neighborhood, and a simulated annealing algorithm that compares candidate networks close to the current network and only accepts a new network if it is better than the current selection ([Bouckaert 1995](#_ENREF_1)).

Support Vector Machine (SVM):

Support vector machine (SVM) approaches construct one or a set of hyperplanes in a high dimensional space optimized to maximize the margin of separation between different classes, and uses the hyperplanes to assign new data points to a target class. SVM has shown superior performance in pattern recognition tasks but is generally computational intensive and is sensitive to parameter settings. Two critical parameters need to be properly tuned to optimize the performance. One parameter is the coefficient affecting the trade-off between errors of SVM on training data and margin maximization, and our tuning range is from 0.01 to 50. The other key parameter is the kernel parameter in constructing non-linear SVM, which is either the exponent that controls the degree of the polynomial kernel and we tune it from 1 to 4, or the gamma parameter that controls the width of the Gaussian Radial Basis Function (RBF) kernel with tuning range from -5 to 3 power of 10.

Random forest:

Random forest classifiers ([Breiman 2001](#_ENREF_2); [Breiman L. 2006](#_ENREF_3)) can outperform other even more sophisticated classifiers, in terms of their performance efficiency and manageability, as well as their relative insensitivity to parameter settings. This method incorporates relationships among attributes since the base learners are classification decision trees. The random forest method uses bootstrap aggregation of classification to reduce bias and cross-validation to assess misclassification rates. It first partitions the dataset into an in-bag set, which uses bootstrap sampling with replacement from the training set, and an out-of-bag set that is set aside to evaluate the model constructed based on in-bag set. It then fits an unpruned decision tree to each bootstrap sample, which is conducted only from a small random subset of variables so the high-dimensionality problem is avoided. The performance of the generated tree model is evaluated using the out-of-bag set recorded as the out-of-bag (oob) error estimate, which provides useful information about the performance of the classifier without touching the testing data set. The value of each variable used in the tree is permuted and its oob value is recorded to compare with the original oob value, and a resulting increase indicates the variable’s importance. From the complete forest, the status of the response variable is predicted as an average or majority vote of the predictions of all trees. Positive importance score indicates a positive correlation with the correct classification. Though the random forest algorithm is relatively less sensitive to parameter setting comparing to other employed classifiers, we use 100 trees to provide the learner a relatively large diverse input set.

Repeat incremental pruning rule learning:

A propositional rule learner using a repeated incremental pruning (RIP) to produce error reduction (RIPPER) is also used in this study. It grows the rule set by greedily adding conditions to the rule until accuracy is maximized, through trying every possible value of each attribute and selecting the condition with highest information gain. Then the algorithm incrementally prunes each rule and computes the smallest possible description length for each variant and the original rule.

Ensemble learning:

Each one of the single classifiers introduced above could be considered as an algorithm for searching through the attribute space to find a model that fits the data, and ensemble learning is a technique to use multiple predictive models in combination in order to achieve superior performance over using a single one. Ensemble methods differ in their base classifiers and the strategies employed in combining base classifiers. We use bagging, boosting, and stacking techniques to combine the predictive model from single classifiers such as tree or Bayesian learners. Bagging, or bootstrap aggregating, randomly takes samples from the training data set with replacement to form a data set and uses it to learn a classifier, repeating this process multiple times and taking votes from constructed models from each iteration with equal weight. The random forest algorithm is, strictly speaking, a bagging ensemble learner with multiple random decision trees as base classifiers. Boosting is different from bagging in that it exerts extra focus on the samples that were misclassified, so that it incrementally constructs a model from every iteration weighting predictions with error from the previous iteration. Stacking combines models constructed from multiple distinct base classifiers. Though ensemble techniques can substantially improve base classifier performance they are computational intensive and are more prone to overfitting.

**Literature Cited:**

Bouckaert, R.R. 1995. *Bayesian Belief Networks: from Construction to Inference.* . Utrecht, Netherlands.

Breiman, L. 2001. Random Forests. *Machine Learning* 45 (1):5-32.

Breiman L., Cutler A., Liaw A, Wiener M *Breiman and Cutler's Random Forests for Classification and Regression.* [R package version 4.5-16]. 2006 [cited. Available from <http://CRAN.R-project.org/package=randomForest>.

Bruner, J., J. J. Goodnow, and G. A. Austin. 1967. *A study of thinking.* , *Science Editions.* New York.

Friedman, N., D. Geiger, and M Goldszmidt. 1997. Bayesian network classifiers. *Maching Learning - Special issue on learning with probabilistic representations* 29:131-163.
