# Supplementary figures and images for: Identification of host-microbe interaction factors in the genomes of soft rot-associated pathogens Dickeya dadantii 3937 and Pectobacterium carotovorum WPP14 with supervised machine learning
Source: BMC Genomics. 2014 Jun 21;15:508. doi: 10.1186/1471-2164-15-508 (PMC4079955; doi:10.1186/1471-2164-15-508)

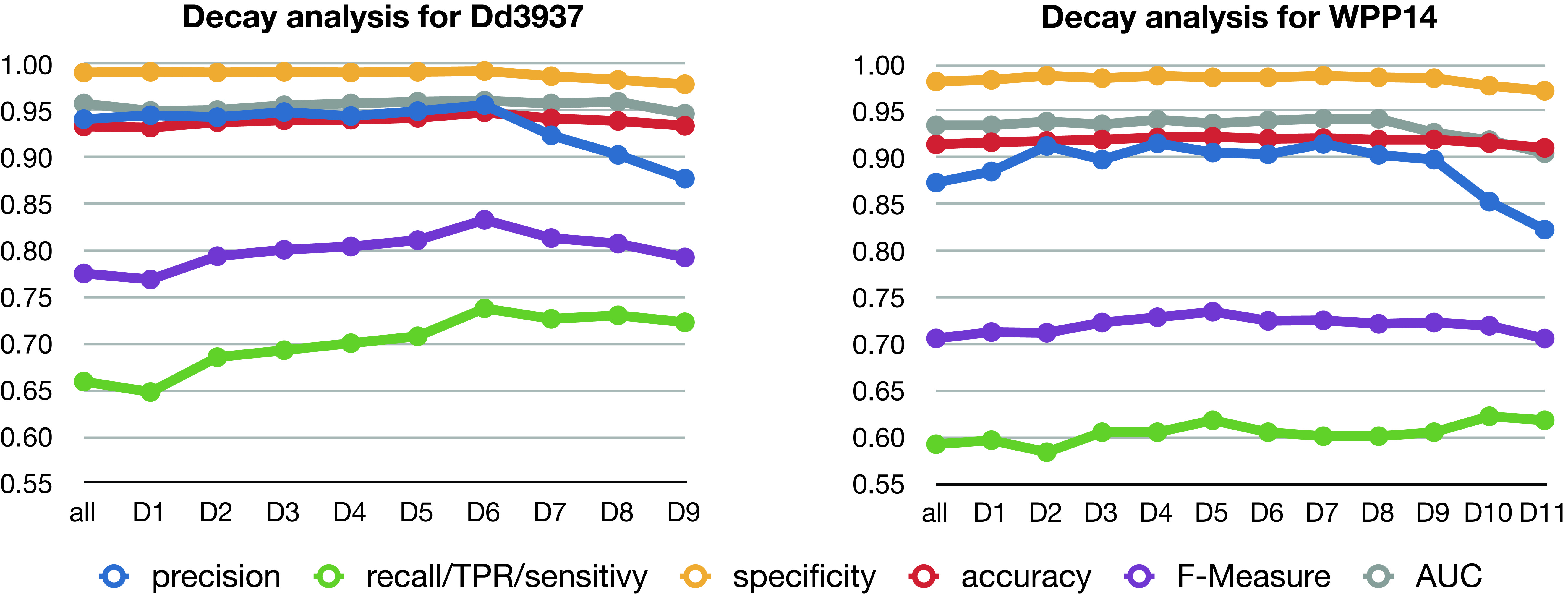

Supplement: Supplementary file 5 — Additional file 5: (a) List of selected attributes with importance measurement score and subsets of attributes defined to be used in data decay analysis for Dickeya dadantii 3937 and Pectobacterium carotovorum WPP14, (b) probability score plot used to define a compact set of attributes for both strains. (ZIP 1 MB) [file 12864_2013_6186_MOESM5_ESM.zip › 2479885711167141_add5b.jpeg]

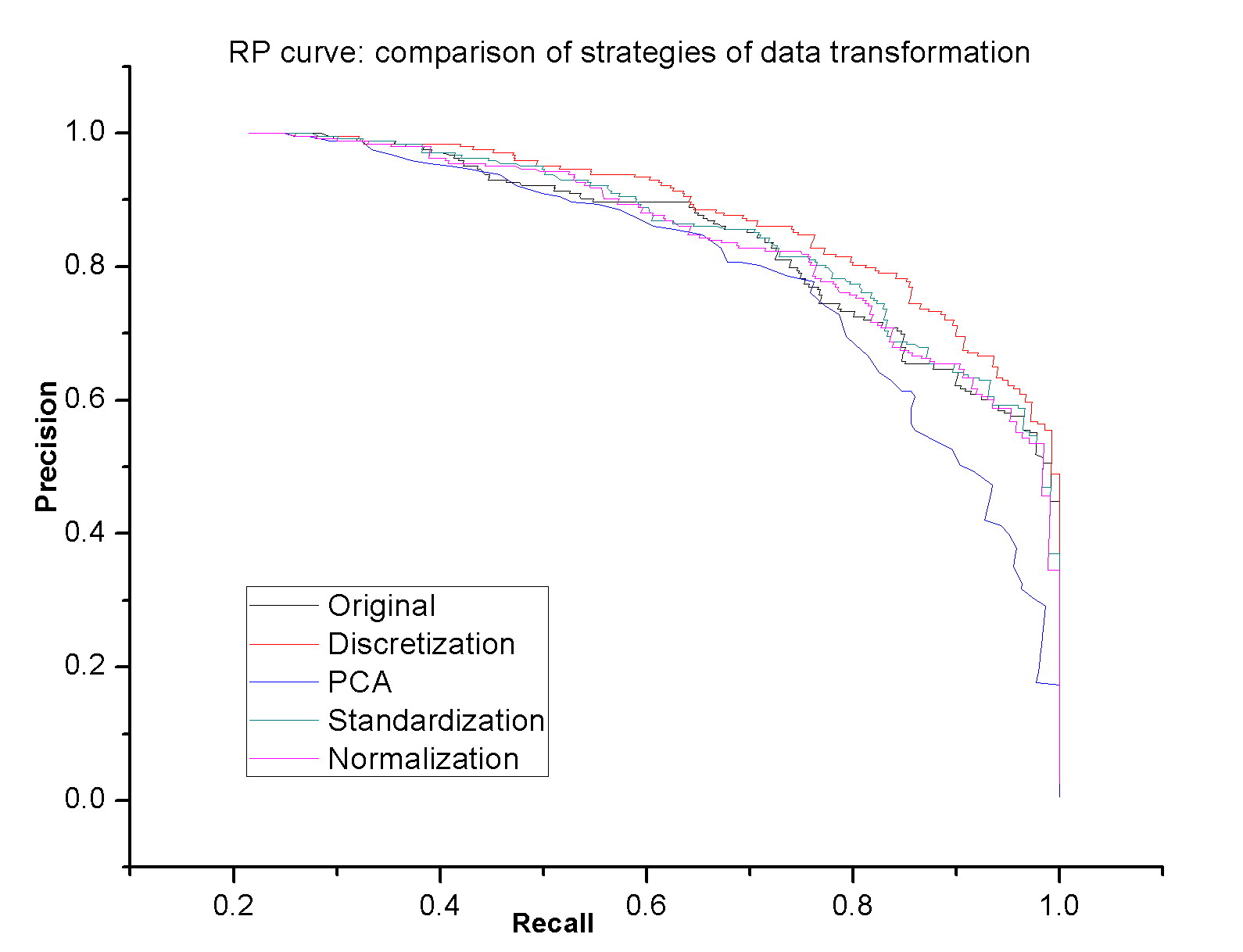

Supplement: Supplementary file 6 — Additional file 6: (a) RP curve to compare strategies of data transformation for boosting classifier performance. (b) PR curve to compare classifier performance using five data sets partitioned according to GO terms for different aspects of host-microbe interaction (refer to Table 2). (ZIP 223 KB) [file 12864_2013_6186_MOESM6_ESM.zip › 2479885711167141_add6a.jpeg]

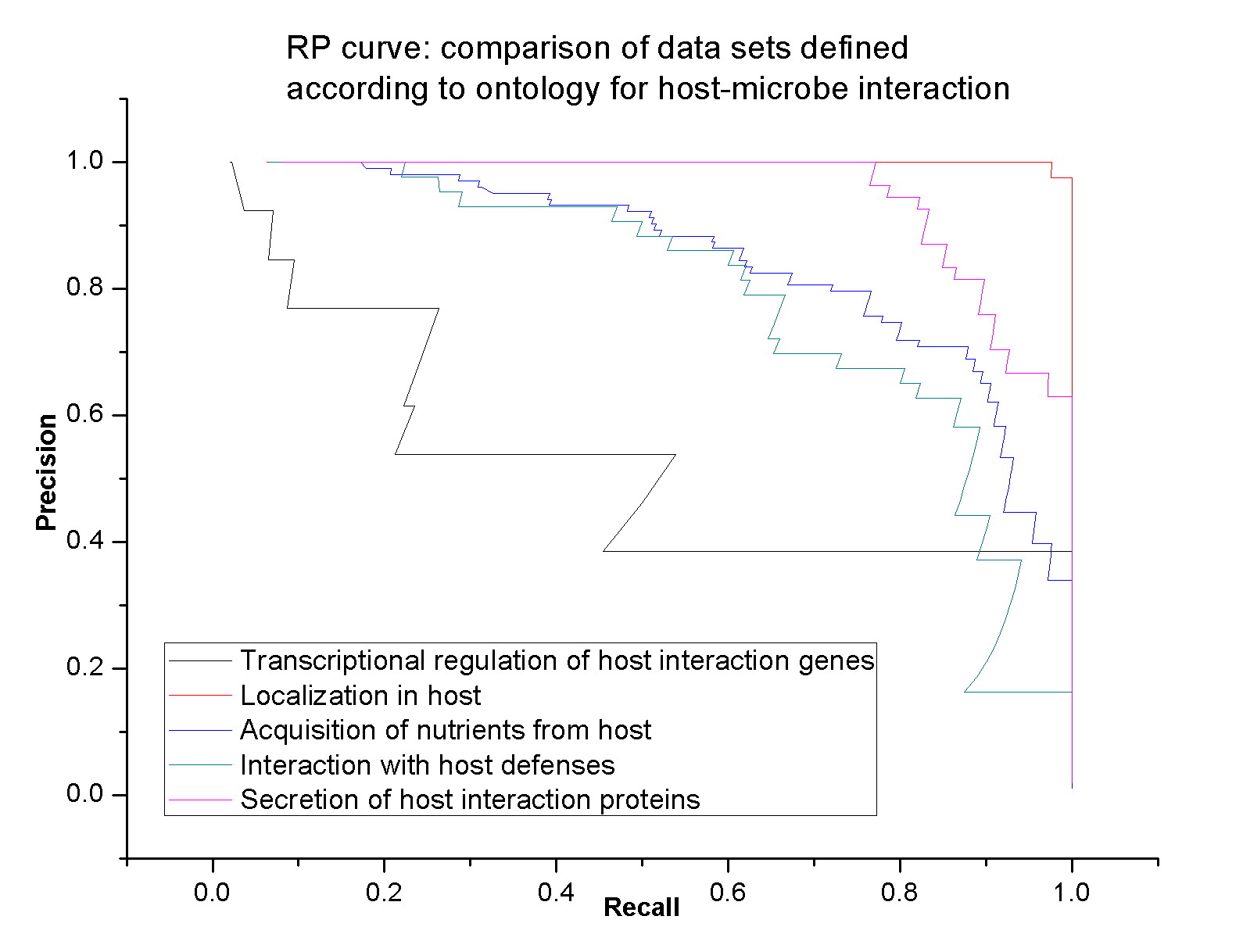

Supplement: Supplementary file 6 — Additional file 6: (a) RP curve to compare strategies of data transformation for boosting classifier performance. (b) PR curve to compare classifier performance using five data sets partitioned according to GO terms for different aspects of host-microbe interaction (refer to Table 2). (ZIP 223 KB) [file 12864_2013_6186_MOESM6_ESM.zip › 2479885711167141_add6b.jpeg]
