# Supplementary material for: Identification of host-microbe interaction factors in the genomes of soft rot-associated pathogens Dickeya dadantii 3937 and Pectobacterium carotovorum WPP14 with supervised machine learning
Source: BMC Genomics. 2014 Jun 21;15:508. doi: 10.1186/1471-2164-15-508 (PMC4079955; doi:10.1186/1471-2164-15-508)
Supplement: Supplementary file 2 — Additional file 2: (a) Strain information of all gamma-proteobacteria with complete or nearly complete genomes used in this study and their associations with various phenotypes of interest (as listed in Additional file 2: Table S1). (b) List of all non gamma-proteobacteria with complete or nearly complete genomes used in this study. (c) List of attribute categories used to generate summary taxonomic and phenotypic attributes based on sequence homology, the count of genomes in each category used as BLASTP search databases, and the number of proteins in Dickeya dadantii (Dd3937) and Pectobacterium carotovorum (WPP14) with hits to target organisms in those categories. (d) List of attributes used in the category of phenotypes of interest, including taxonomic groups and lifestyle statistics. (ZIP 223 KB) [file 12864_2013_6186_MOESM2_ESM.zip › 2479885711167141_add2c.doc]

Table S1. List of attribute categories used to generate summative taxonomic and phenotypic attributes based on sequence homology in this study, and number of attributes in each categories for all data points in training data set for *Dickeya dadantii* (Dd3937) and *Pectobacterium carotovorum* (WPP14)

|  | **Category** | **Count** | **Dd3937** | **WPP14** |
| --- | --- | --- | --- | --- |
| **Taxonomy** | Enterobacteriales | 106 | 4042 | 4033 |
|  | *Pseudomonadales* | 30 | 3043 | 2904 |
|  | *Alteromonadales* | 27 | 3112 | 3010 |
|  | *Pasteurellales* | 15 | 1782 | 1771 |
|  | *Xanthomonadales* | 14 | 1757 | 1689 |
|  | *Vibrionales* | 13 | 2347 | 2327 |
|  | *Thiotrichales* | 10 | 1241 | 1184 |
|  | *Legionellales* | 9 | 1142 | 1119 |
|  | Gamma strains | 239 | 4088 | 4103 |
|  | Non-gamma strains | 58 | 3007 | 2861 |
|  | Endosymbionts | 11 | 1017 | 1010 |
|  | Unique data points* | - | 432 | 487 |
| Growth | Facultative | 160 | 4059 | 4056 |
|  | Aerobic | 72 | 3587 | 3501 |
|  | Anaerobic | 7 | 2836 | 2836 |
| **Habitat** | Aquatic | 31 | 3241 | 3139 |
|  | Host-associated | 83 | 3599 | 3580 |
|  | Multiple | 104 | 4056 | 4034 |
|  | Specialized | 21 | 3168 | 3039 |
| **Pathogenicity** | Animal-associated | 140 | 3721 | 3736 |
|  | Non-pathogenic | 67 | 3657 | 3568 |
|  | Plant-associated | 32 | 3978 | 3931 |

*Unique data points are defined as the genes that do not any BLAST reciprocal best hit to any Gamma-proteobacterium.
