# Supplementary material for: Identification of host-microbe interaction factors in the genomes of soft rot-associated pathogens Dickeya dadantii 3937 and Pectobacterium carotovorum WPP14 with supervised machine learning
Source: BMC Genomics. 2014 Jun 21;15:508. doi: 10.1186/1471-2164-15-508 (PMC4079955; doi:10.1186/1471-2164-15-508)
Supplement: Supplementary file 3 — Additional file 3: Functional genomics data sets used in this study. (a) Data sets generated from microarray or proteomics studies. (b) Binding sites prediction of 32 putative transcriptional factors that may be related with host-microbe interactions. (ZIP 34 KB) [file 12864_2013_6186_MOESM3_ESM.zip › 2479885711167141_add3a.doc]

**Supplemental File 3.** Functional genomics data used in this study

(a) Microarray and proteomics data sets used in this study. The source of original and processed data set is listed in table. The log2ratio or experimental condition and control condition in each of the experiment was used in this study (if not obtainable from the downloaded dataset, the log2ratio was calculated subsequently).

*denote the experiment performed on different organism than the target organisms, in such case the reciprocal best hit between experiment organism and the target organisms was used so that the expression value can be connected to target organisms.

| **Organisms** | **Brief description of experiments** | **Citation or GEO (Gene Expression OmniBus) Accession No.** |
| --- | --- | --- |
| Dd3937 | PhoP mutant |  |
|  | PH acid stress |  |
|  | Jasmonic acid response |  |
|  | TH response |  |
|  | Aerobic/Anaerobic shift |  |
|  | PecS mutant | E-TABM-428 |
|  | Potato juice response | Unpublished data from Dr. Perna laboratory (ntperna@wisc.edu) |
|  | African violet leaves |  |
|  | PhoQ |  |
|  | Phenolic acid response | GSE4558 |
|  | YpCO92 plague isolates* | GSE3575 |
|  | *LuxS* mutants* |  |
|  | Carbonate insoluble outer membrane fraction |  |
| WPP14 | SCRI1043-potato tuber/stem isolates* | GSE11548 |
|  | SCRI1043-DsbA* | GSE10647 |

Reference used in Supplemental File 3a

1.	Rodriguez-Palenzuela P, Matas IM, Murillo J, Lopez-Solanilla E, Bardaji L, Perez-Martinez I, Rodriguez-Moskera ME, Penyalver R, Lopez MM, Quesada JM *et al*: **Annotation and overview of the Pseudomonas savastanoi pv. savastanoi NCPPB 3335 draft genome reveals the virulence gene complement of a tumour-inducing pathogen of woody hosts**. *Environ Microbiol* 2010, **12**(6):1604-1620.

2.	Babujee L, Apodaca J, Balakrishnan V, Liss P, Kiley PJ, Charkowski AO, Glasner JD, Perna NT: **Evolution of the metabolic and regulatory networks associated with oxygen availability in two phytopathogenic enterobacteria**. *BMC Genomics* 2012, **13**:110.

3.	Okinaka Y, Yang C-H, Perna N, Keen NT: **Microarray profiling of *Erwinia chrysanthemi* 3937 genes that are regulated during plant infection.** *Mol Plant-Microbe Interact* 2002, **15**(7):619-629.

4.	Venkatesh B, Babujee L, Liu H, Hedley P, Fujikawa T, Birch P, Toth I, Tsuyumu S: **The Erwinia chrysanthemi 3937 PhoQ sensor kinase regulates several virulence determinants**. *Journal of Bacteriology* 2006, **188**(8):3088-3098.

5.	Coulthurst SJ, Lilley KS, Salmond GPC: **Genetic and proteomic analysis of the role of luxS in the enteric phytopathogen, Erwinia carotovora**. *Molecular Plant Pathology* 2006, **7**(1):31-45.

6.	Babujee L, Venkatesh B, Yamazaki A, Tsuyumu S: **Proteomic analysis of the carbonate insoluble outer membrane fraction of the soft-rot pathogen Dickeya dadantii (syn. Erwinia chrysanthemi) strain 3937**. *Journal of Proteome Research* 2007, **6**(1):62-69.
