# Supplementary material for: Identification of host-microbe interaction factors in the genomes of soft rot-associated pathogens Dickeya dadantii 3937 and Pectobacterium carotovorum WPP14 with supervised machine learning
Source: BMC Genomics. 2014 Jun 21;15:508. doi: 10.1186/1471-2164-15-508 (PMC4079955; doi:10.1186/1471-2164-15-508)
Supplement: Supplementary file 3 — Additional file 3: Functional genomics data sets used in this study. (a) Data sets generated from microarray or proteomics studies. (b) Binding sites prediction of 32 putative transcriptional factors that may be related with host-microbe interactions. (ZIP 34 KB) [file 12864_2013_6186_MOESM3_ESM.zip › 2479885711167141_add3b.doc]

| Index | Binding Site name | virulence | HMM Cut-off score | #Binding Site | Upstream/downstream | Reference |
| --- | --- | --- | --- | --- | --- | --- |
| 1 | kdgR | Y | 12 | 131 | -300+50 |  |
| 2 | FNR | N | 10 | 401 | -200+50 |  |
| 3 | pecS | Y | 12 | 39 | -320+200 |  |
| 4 | hrpL | Y | 12 | 25 | -220+150 |  |
| 5,6 | CRP | Y | 10/12 | 300/211 | -320+50 |  |
| 7 | ExuR/UxuR | Y | 12 | 56 | -300+50 |  |
| 8 | GntR | N | 12 | 63 | -250+50 |  |
| 9 | fur | Y | 15 | 78 | -320+50 |  |
| 10 | H-NS | Y | 12 | 110 | -300+100 |  |
| 11 | IscR-type 1 | Y | 11 | 29 | -260+50 |  |
| 12 | IscR-type 2 | Y | 14 | 17 | -250+50 |  |
| 13 | FruR/Cra | N | 13 | 37 | -220+50 |  |
| 14 | GalR/GalS | N | 13 | 20 | -400+50 |  |
| 15 | lacI | N | 12 | 19 | -400+50 |  |
| 10 | H-NS | Y | 12 | 110 | -300+100 |  |
| 16 | RbsR | N | 15 | 11 | -400+50 |  |
| 17 | PurR | N | 13 | 75 | -400+200 |  |
| 18 | RafR | N | 14 | 21 | -400+50 |  |
| 19 | ScrR | N | 14 | 15 | -400+50 |  |
| 20 | arcA | N | 14 | 15 | -400+50 |  |
| 21 | ascG | N | 13 | 14 | -400+50 |  |
| 22 | ECA2487 | N | 15 | 7 | -400+50 |  |
| 23 | ECA3649 | N | 11 | 38 | -400+50 |  |
| 24 | NadR | N | 15 | 14 | -400+50 |  |
| 25 | ModE | N | 15 | 22 | -300+50 |  |
| 26 | NagC | N | 16 | 44 | -300+50 |  |
| 27 | PdhR | N | 13 | 6 | -400+50 |  |
| 28 | RKP05499 | N | 20 | 2 | -400+50 |  |
| 29 | STM3696 | N | 17 | 3 | -400+50 |  |
| 30 | XylR | N | 26 | 2 | -300+50 |  |
| 31 | YPO0846 | N | 16 | 14 | -400+50 |  |
| 32 | YPO2568 | N | 14 | 3 | -400+50 |  |

**Supplemental File 3b.** List of the binding site prediction used in this study. It is based on a training set of known transcription factor (TF) binding site sequences (obtained from RegulonDB ), subsequent modeling and prediction procedures for each individal TF was implemented in HMMER package based on Hidden Markov Models (HMMs).

Reference used in Supplemental file 3b:

1.	Rodionov DA, Gelfand MS, Hugouvieux-Cote-Pattat N: Comparative genomics of the KdgR regulon in *Erwinia chrysanthemi* 3937 and other gamma-proteobacteria. *Microbiol* 2004, 150:3571-3590.

2.	Compan I, Touati D: Anaerobic activation of arcA transcription in Escherichia coli: roles of Fnr and ArcA. *Mol Microbiol* 1994, 11(5):955-964.

3.	Rouanet C, Reverchon S, Rodionov DA, Nasser W: Definition of a consensus DNA-binding site for PecS, a global regulator of virulence gene expression in Erwinia chrysanthemi and identification of new members of the PecS regulon. *J Biol Chem* 2004, 279(29):30158-30167.

4.	Yang S, Peng Q, Zhang Q, Zou L, Li Y, Robert C, Pritchard L, Liu H, Hovey R, Wang Q *et al*: Genome-wide identification of HrpL-regulated genes in the necrotrophic phytopathogen Dickeya dadantii 3937. *PLoS One* 2010, 5(10):e13472.

5.	Rodionov DA, Mironov AA, Rakhmaninova AB, Gelfand MS: Transcriptional regulation of transport and utilization systems for hexuronides, hexuronates and hexonates in gamma purple bacteria. *Mol Microbiol* 2000, 38(4):673-683.

6.	Gama-Castro S, Salgado H, Peralta-Gil M, Santos-Zavaleta A, Muniz-Rascado L, Solano-Lira H, Jimenez-Jacinto V, Weiss V, Garcia-Sotelo JS, Lopez-Fuentes A *et al*: RegulonDB version 7.0: transcriptional regulation of Escherichia coli K-12 integrated within genetic sensory response units (Gensor Units). *Nucleic Acids Res* 2011, 39(Database issue):D98-105.

7.	Chen Z, Lewis KA, Shultzaberger RK, Lyakhov IG, Zheng M, Doan B, Storz G, Schneider TD: Discovery of Fur binding site clusters in Escherichia coli by information theory models. *Nucleic Acids Res* 2007, 35(20):6762-6777.

8.	Lavrrar JL, McIntosh MA: Architecture of a fur binding site: a comparative analysis. *J Bacteriol* 2003, 185(7):2194-2202.

9.	Lang B, Blot N, Bouffartigues E, Buckle M, Geertz M, Gualerzi CO, Mavathur R, Muskhelishvili G, Pon CL, Rimsky S *et al*: High-affinity DNA binding sites for H-NS provide a molecular basis for selective silencing within proteobacterial genomes. *Nucleic Acids Res* 2007, 35(18):6330-6337.

10.	Nesbit AD, Giel JL, Rose JC, Kiley PJ: Sequence-specific binding to a subset of IscR-regulated promoters does not require IscR Fe-S cluster ligation. *J Mol Biol* 2009, 387(1):28-41.

11.	Giel JL, Rodionov D, Liu M, Blattner FR, Kiley PJ: IscR-dependent gene expression links iron-sulphur cluster assembly to the control of O2-regulated genes in Escherichia coli. *Mol Microbiol* 2006, 60(4):1058-1075.

12.	Eddy SR: Profile hidden Markov models. *Bioinformatics* 1998, 14(9):755-763.
